# Structural Analysis of the cl-Par-4 Tumor Suppressor as a Function of Ionic Environment

**DOI:** 10.3390/biom11030386

**Published:** 2021-03-05

**Authors:** Krishna K. Raut, Komala Ponniah, Steven M. Pascal

**Affiliations:** Department of Chemistry and Biochemistry, Old Dominion University, Norfolk, VA 23529, USA; kraut@odu.edu (K.K.R.); kponniah@odu.edu (K.P.)

**Keywords:** intrinsically disordered protein (IDP), prostate apoptosis response-4 (Par-4), tumor suppressor, circular dichroism (CD) spectroscopy, dynamic light scattering (DLS), aggregation, coiled-coil, caspase

## Abstract

Prostate apoptosis response-4 (Par-4) is a proapoptotic tumor suppressor protein that has been linked to a large number of cancers. This 38 kilodalton (kDa) protein has been shown to be predominantly intrinsically disordered in vitro. In vivo, Par-4 is cleaved by caspase-3 at Asp-131 to generate the 25 kDa functionally active cleaved Par-4 protein (cl-Par-4) that inhibits NF-κB-mediated cell survival pathways and causes selective apoptosis in tumor cells. Here, we have employed circular dichroism (CD) spectroscopy and dynamic light scattering (DLS) to assess the effects of various monovalent and divalent salts upon the conformation of cl-Par-4 in vitro. We have previously shown that high levels of sodium can induce the cl-Par-4 fragment to form highly compact, highly helical tetramers in vitro. Spectral characteristics suggest that most or at least much of the helical content in these tetramers are non-coiled coils. Here, we have shown that potassium produces a similar effect as was previously reported for sodium and that magnesium salts also produce a similar conformation effect, but at an approximately five times lower ionic concentration. We have also shown that anion identity has far less influence than does cation identity. The degree of helicity induced by each of these salts suggests that the “Selective for Apoptosis in Cancer cells” (SAC) domain—the region of Par-4 that is most indispensable for its apoptotic function—is likely to be helical in cl-Par-4 under the studied high salt conditions. Furthermore, we have shown that under medium-strength ionic conditions, a combination of high molecular weight aggregates and smaller particles form and that the smaller particles are also highly helical, resembling at least in secondary structure, the tetramers found at high salt.

## 1. Introduction

Prostate apoptosis response-4 (Par-4) is a tumor suppressor protein that is capable of selectively inducing apoptosis in cancer cells while leaving healthy cells unaffected [1]. Par-4 was first identified through its down-regulation in therapy-resistant prostate cancer cells and has since been shown to be ubiquitously expressed, localized in the cytoplasm, the nucleus and extracellularly, and linked to a variety of cancers [2,3,4,5]. The *par-4* gene maps to human chromosome 12q21 and encodes an approximately 40 kilodalton (kDa) protein of 340 amino acids [6]. While Par-4 has been shown to be largely intrinsically disordered in vitro, it does contain several identifiable domains and features. The first identified domain was the C-terminal coiled-coil, which is highly conserved across humans, mice, and rats [7]. This region contains a small nuclear export signal (NES). The coiled-coil is responsible for Par-4 dimerization, and for most known interactions with cellular proteins. Two nuclear localization signals are found in the N-terminal half of the protein, though only the 2nd, NLS2, has been shown to affect localization [8,9]. Par-4 also contains a unique and highly conserved “Selective for Apoptosis in Cancer cells” (SAC) domain, which is the minimal region required to induce apoptosis [8,9].

Although Par-4 is expressed in both normal and cancer cells, only cancer cells are typically susceptible to the killing effect of this protein. The two best-documented factors controlling this selectivity are (i) GRP78 levels and (ii) PKA levels. (i) Extra-cellular Par-4 can enter cancer cells by binding to the surface receptor GRP78 via the Par-4 SAC domain [10]. GRP78 levels in healthy cells are typically very low or even undetectable, abrogating entry of extracellular Par-4 into healthy cells. (ii) Protein Kinase A (PKA) is required to phosphorylate intracellular Par-4 at residue T163, which is within the SAC domain [4,11,12]. This phosphorylation event leads to the inhibition of PKC-ζ by Par-4, which in turn suppresses phosphorylation of FADD by PKC-ζ. Unphosphorylated FADD is required for DISC (Death-Inducing Signal Complex) formation at the plasma membrane, which triggers the caspase-8-mediated apoptotic pathway [9,10]. As with GRP78, the basal level of PKA in healthy cells is typically low and insufficient to cause phosphorylation of Par-4, thereby preventing DISC formation in healthy cells.

Interestingly, one key event downstream of caspase-8 activation is the activation of caspase-3, which in turn cleaves intracellular Par-4. Caspase-3 cleaves Par-4 after D131, producing two fragments: a 15 kDa amino-terminal fragment (called PAF for Par-4 Amino terminal Fragment) and a 25 kDa carboxy-terminal fragment (called cl-Par-4 for cleaved) [8,13]. Assisted by NLS2, cl-Par-4 translocates to the nucleus, where it blocks cell survival pathways by inhibiting transcriptional regulators such as NF-κB [14,15].

Intrinsically disordered proteins (IDPs) or intrinsically disordered protein regions (IDPRs) possess specific features such as a high percentage of charged residues and a low percentage of hydrophobic amino acids [16,17,18]. These exceptional characteristics lead to a disordered conformation under typical physiological conditions [19]. A number of environmental factors can, however, influence the folding of these proteins including temperature, pH, ionic strength, post-translational modification including both phosphorylation and targeted cleavage by proteases, and interaction with natural ligands or protein partners [16,20,21]. Significant evidence has arisen showing enhanced folding of IDPs with increased ion concentration and ionic charge density, though unfolding can also be triggered by the same factors [22].

We have previously shown that high levels of sodium chloride or acidic pH can induce the cl-Par-4 fragment to form highly compact, highly helical structures in vitro, at high micromolar protein concentrations [23,24]. The ionic conditions required to favor these small, well-folded particles are outside of the range of conditions normally found in cells. This led to the hypothesis that multiple factors, including multiple ion types, may combine forces to influence Par-4 conformation. To begin to test this hypothesis, we here have examined and compared the structural characteristics of cl-Par-4 as a function of sodium chloride, potassium chloride, magnesium chloride, and magnesium sulfate concentration. The results shed light on the role of cations versus anions, their charge densities, and also on the role of the caspase-induced cleavage event that produces cl-Par-4, in influencing Par-4 structure, and subsequently, Par-4 localization and function.

## 2. Materials and Methods

### 2.1. Expression and Purification of cl-Par-4

BL21 (DE3) *Escherichia coli* cells transfected with a codon-optimized construct of cl-Par-4 in the modified expression vector H-MBP-3C [25] were grown in Luria-Bertani (LB) media with 100 µg/mL ampicillin at 37 °C until an OD_600_ of 0.8–0.9 was reached. The cells were then induced for protein expression with the addition of 0.5 mM isopropyl thio-β-D-galactoside (IPTG) and grown at 15 °C until OD_600_ reached 1.5–1.6. The harvested cells were lysed by a combination of mechanical (sonication) and enzymatic (lysozyme) methods and extracted protein was purified with immobilized metal affinity chromatography (IMAC) using a HisTrap HP 5 mL column (GE Healthcare, Uppsala, Sweden). The His-MBP tag was cleaved from cl-Par-4 by treating the protein with the His-tagged 3C-protease enzyme. The purified protein was then further dialyzed against the storage buffer (10 mM Tris, 1 M NaCl, 1 mM TCEP, pH 7.0) and concentrated by centrifugation using a 10 kDa MWCO Vivaspin Turbo 15 (Sartorius, Epsom, UK). The protein concentration was determined by taking absorbance readings at 280 nm using a BioDrop DUO (BioDrop Ltd., Cambridge, UK) and the theoretical extinction coefficient of 6400 M^−1^cm^−1^. The protein was finally lyophilized using a FreeZone Freeze Dryer (LABCONCO Corporation, Kansas City, MO, USA), stored at −80 °C, and re-solubilized in sterile distilled water when required. In our experience, in vitro CD and DLS data obtained with cl-Par-4 samples prepared, lyophilized, and redissolved as described above were indistinguishable from data obtained with fresh (never lyophilized) samples.

### 2.2. Circular Dichroism Spectroscopy

Circular dichroism (CD) spectra of the protein were obtained on a J-815 CD spectrometer (Jasco, Easton, MD, USA). The cl-Par-4 samples were at a concentration of 0.2 mg/mL in Tris-HCl buffer (10 mM Tris, 1 mM DTT, pH 7.0) with varying concentrations of monovalent and divalent cations present (10–1000 mM). Each cl-Par-4 sample used for CD spectroscopy contained 40 mM NaCl as a residual due to the dilution from the storage buffer (10 mM Tris, 1 mM TCEP, and 1 M NaCl, pH 7.0). For the filtered samples, sample preparation was identical except for the filtration of the sample through a 0.45-micron Nalgene SFCA membrane syringe filter (Thermo Fisher Scientific, Rochester, NY, USA) immediately prior to CD spectroscopy. For the centrifuged samples, sample preparation was identical except that samples were centrifuged for 5 min at 10,000 RPM (9615× *g*) using a Sorvall Legend Micro 21R Centrifuge (Thermo Fisher Scientific, Germany), and the supernatant was used for the CD spectroscopy. CD data were recorded over a 400–190 nm wavelength range at a scan speed of 20 nm/min using a 1 mm pathlength quartz cuvettes at 25 °C. A buffer blank was subtracted from the spectra, which were then smoothed using a means-movement function of 25 nm and converted to molar ellipticity The deconvolution of CD spectra for estimation of secondary structure content was done using the SELCON3 algorithm through the DichroWeb server [26].

### 2.3. Dynamic Light Scattering

Dynamic light scattering (DLS) data were recorded using a NanoBrook Omni particle sizer and zeta potential analyzer (Brookhaven Instruments Corporation, Holtsville, NY, USA). Protein samples were at a concentration of 0.2 mg/mL in Tris buffer (10 mM Tris, 1 mM DTT, pH 7.0) with varying concentrations of monovalent and divalent cations (10–1000 mM). Filtered and centrifuged samples were prepared as described above. The data were recorded at 25 °C using a standard diode laser at 640 nm wavelength, scattering angle of 173°, and plastic cuvettes of 1 cm pathlength. Five scans were recorded for each sample and hydrodynamic radii (Stokes’ radii) were calculated from the mean effective diameter obtained from the summary statistical report of the NanoBrook software.

### 2.4. SDS-PAGE

Sodium dodecyl sulfate-polyacrylamide gel electrophoresis (SDS-PAGE) gels of 4–12% were prepared using a gel casting system (Bio-Rad Laboratories, Inc., Irvine, CA, USA). Filtered samples were prepared as mentioned above. The samples, each containing initially 0.2 mg/mL of the protein (filtered samples contained less protein), were prepared for loading by combining 16 µL of each sample with 4 µL of 5× sample loading dye, mixed well, and heated at 90 °C for 2 min. Next, 20 µL of each sample was loaded into separate wells in the gel. The gels were run in 1× SDS-PAGE buffer (Tris base, glycine, SDS, and water) using the PowerPac Basic device (Bio-Rad Laboratories, Inc., Irvine, CA, USA) for 20 min with a low voltage of 50 V and then 60 min with a high voltage of 150 V. The gels were then stained for 5 h in Coomassie stain (0.2% R-250, 7.5% acetic acid, 50% ethanol and water) and de-stained overnight in de-staining solution (7.5% acetic acid, 50% ethanol, and water). Visualization of the gel was done on a white lightbox.

## 3. Results

### 3.1. Disorder Prediction in cl-Par-4

**Figure 1:**
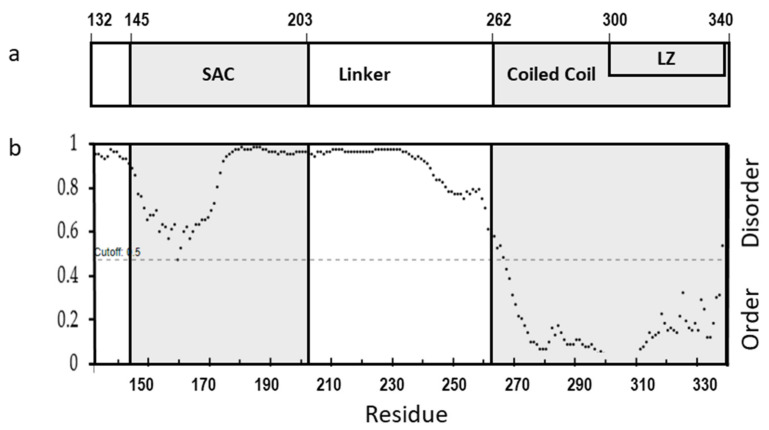
(**a**) Schematic diagram showing domains of cleaved Par-4 protein (cl-Par-4) and (**b**) Disorder prediction in cl-Par-4 using DISOPRED3.

The cl-Par-4 fragment is the functionally active fragment of the Par-4 protein that enters the nucleus. This region of the protein can be further subdivided as follows: (Figure 1a): the SAC (Selective for Apoptosis induction in Cancer cells) domain, the CC (Coiled-Coil) domain, and the Linker between these two domains. In addition, the SAC domain contains an NLS (Nuclear Localization Signal) near its N-terminus, and the CC domain contains an LZ (Leucine Zipper) domain which comprises its C-terminal half [23]. Prediction of disorder in these domains was done by using DISOPRED3, which utilizes X-ray diffraction databases (Figure 1b) [27,28]. Considering a probability of 0.5 (dashed line) as the demarcation line, the analysis clearly predicts order in the CC domain and disorder in the Linker, while the SAC domain has intermediate character, approaching the order/disorder line. Previous sequence analysis also showed mixed order/disorder propensity and some helical character in the SAC domain, and so order in the SAC domain would not be surprising, under the right conditions [23].

### 3.2. Differential Effect of Monovalent & Divalent Ions on the Structure of cl-Par-4

**Figure 2:**
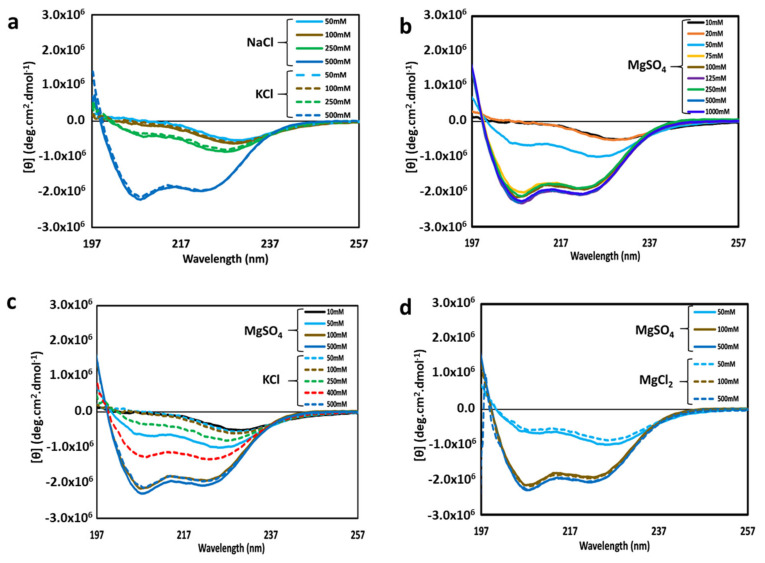
Overlay of circular dichroism (CD) spectra of cl-Par-4 at various concentrations of the following salts: (**a**) NaCl (solid lines) and KCl (dashed lines); (**b**) MgSO_4_; (**c**) MgSO_4_ (solid lines) and KCl (dashed lines); (**d**) MgSO_4_ (solid lines) and MgCl_2_ (dashed lines). The salt concentrations are indicated by the color legend within each panel. All spectra were recorded at pH 7.0.

We have previously shown that at a high NaCl concentration, cl-Par-4 forms a highly helical tetramer with mostly non-coiled coil helices [24]. Here, we investigated the effects of high levels of a different monovalent cation (potassium), with a different size and charge density. The relative effects of NaCl and KCl on CD spectra of cl-Par-4 are shown in Figure 2a. All cl-Par-4 samples were at a concentration of 0.2 mg/mL in Tris-buffer of pH 7.0 containing various concentrations of either NaCl or KCl.

At a 500 mM salt concentration, CD spectra were characteristic of α-helical content with intense dichroism minima at 222 nm and 208 nm [29,30]. At lower salt concentrations, the intensity of the CD spectra was reduced, which will be discussed further in Section 3.4. At all concentrations, the effect of Na vs. K cations appeared to be similar.

For isolated alpha-helices, an intensity ratio of greater than 1 for the two minima at θ_222_ and θ_208_ indicates coiled-coil formation [24,29,31]. By these criteria, it appears that the 500 mM salt sample did not form a significant coiled-coil, but that the lower salt samples may. An alternative interpretation of conformation at low and medium salt concentrations is presented in the discussion section.

The effects of the divalent cation magnesium on the structural conformation of cl-Par-4 were also investigated via CD spectroscopy. Sample preparation was similar to above, but with various concentrations of MgSO_4_ instead of NaCl or KCl. CD spectra (Figure 2b) showed a similar trend as obtained with monovalent cations, with higher intensity at higher salt concentration. Again, the θ_222_: θ_208_ ratio suggests little coiled-coil formation at high salt concentration and the possibility of coiled-coil at low salt concentration.

However, the most striking difference between monovalent and divalent cation salt samples was in the concentrations of demarcation: all samples with 75 mM and higher MgSO_4_ showed similar, intense, highly helical but non-coiled coil CD spectra, whereas only the 500 mM monovalent salt samples produced such a result. Intermediate spectra occurred for 50 mM MgSO_4_, while analogous intermediate spectra occurred at a much higher monovalent salt concentration of 250 mM NaCl or KCl. The effects of MgSO_4_ and KCl are directly compared in Figure 2c. These results suggest that the divalent magnesium cation is five or more times as effective as monovalent cations in influencing the structure of cl-Par-4.

Next, the effect of monovalent versus divalent anions on the structure of cl-Par-4 was investigated by comparing samples prepared with MgSO_4_ versus MgCl_2_. The results showed little difference between like concentrations of MgSO_4_ and MgCl_2_ (Figure 2d), suggesting that cation identity exerts a far greater influence on cl-Par-4 structure than does anion identity.

### 3.3. Effect of Monovalent & Divalent Ions on Hydrodynamic Size of cl-Par-4

**Figure 3:**
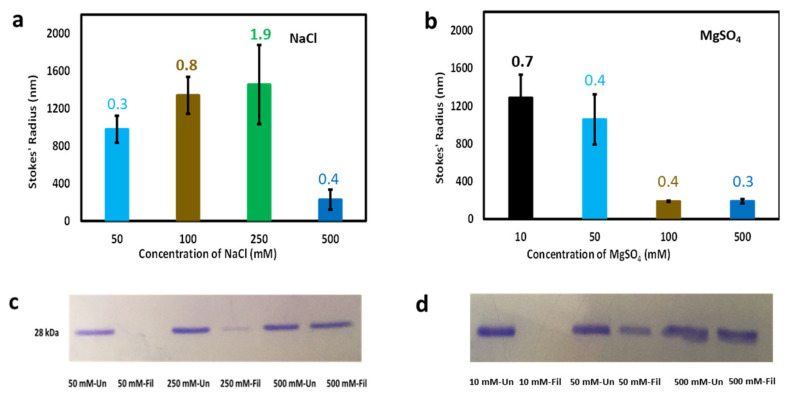
Particle size analysis of cl-Par-4 as a function of salt concentration. (**a**) dynamic light scattering (DLS)-derived Stokes’ radii vs. NaCl concentration. (**b**) DLS-derived Stokes’ radii vs. MgSO_4_ concentration. (**c**) SDS-PAGE analysis of samples from panel (**a**), before and after filtration. (**d**) SDS-PAGE analysis of samples from panel (**b**), before and after filtration. In panels (**a**) and (**b**), error bars represent the standard deviation in Stokes’ radii from multiple runs, and the number above each bar represents the average polydispersity value for these runs.

The size of protein molecules in solution (hydrodynamic size) provides information about conformation. Small size indicates a compact and well-folded conformation whereas large size indicates a highly self-associated, or aggregated conformation, that may or may not also be significantly disordered. The hydrodynamic size distribution of cl-Par-4 was investigated using dynamic light scattering (DLS). Sample preparation was similar to above, but with the following concentrations of NaCl: 50, 100, 250, and 500 mM or MgSO_4_: 10, 50, 100, and 500 mM. The result showed relatively small particles (Stokes’ radius, Rs ≈ 200 nm) in the sample with 500 mM of NaCl (Figure 3a). The observed hydrodynamic sizes were significantly larger (Rs ≈ 1000 nm) at the three lower NaCl concentrations (50, 100, and 250 mM), indicating the presence of large aggregates. The polydispersity values associated with DLS measurements (see numbers above bars in Figure 3a) correlate strongly with Stokes’ radii, indicating that in general, samples with larger particles also contain a broader range of particle sizes.

A similar trend of smaller particle size at higher salt was observed in the presence of MgSO_4_ (Figure 3b). However, the transition between small (Rs ≈ 200 nm) and large (Rs ≈ 1000 nm) particles apparently occurs somewhere between MgSO_4_ concentrations of 50 mM and 100 mM, suggesting that the divalent magnesium ion is approximately five times more potent than the monovalent cation in influencing cl-Par-4 particle size. Again, the largest Stokes’ radii (at 10 mM MgSO_4_) were associated with the broadest range of particle sizes, as shown by polydispersity measurements in Figure 3b. However, in general, less polydispersity was seen in the MgSO_4_ samples than in the NaCl samples, indicating that even when large particles were present, magnesium induced a more uniform particle size than did sodium.

Note that large particle size as detected by DLS (Figure 3a,b) corresponded closely with reduced CD intensity (Figure 2a,b). However, the correspondence was not perfect. In particular, note that DLS detected similar particle sizes at 50, 100, and 250 mM NaCl (Figure 3a), but that 250 mM monovalent salt produced a significantly higher dichroism intensity than 50 or 100 mM had (Figure 2a). The MgSO_4_ data was more self-consistent, but still not perfectly so, particularly at 50 mM (see Figure 2b and Figure 3b). This inconsistency with the intermediate concentrations of both monovalent and divalent cations could be due to the fact that scattering is highly sensitive to the largest particles in solution. The result was consistent with the concept that the largest particles were as shown in Figure 3a,b, but that more of these large particles form at lower salt concentrations, which resulted in more CD signal loss, possibly due at least in part to a smaller effective cross-section being available for CD measurements [32].

Sample filtration was next used to further analyze the relationship between CD spectroscopy and particle size. The fraction of protein removed by filtration was analyzed via SDS-PAGE. For this analysis, 0.2 mg/mL cl-Par-4 samples with low salt (50 mM NaCl or 10 mM MgSO_4_), intermediate salt (250 mM NaCl or 50 mM MgSO_4_), and high salt (500 mM NaCl or 500 mM MgSO_4_) were used. Corresponding filtered samples were prepared by passing the samples through 0.45-micron filters. The results for NaCl and MgSO_4_ samples are shown in Figure 3c,d, respectively. Each of the unfiltered samples showed similar gel band intensity. After filtration, the bands for low salt samples essentially disappeared, the bands for medium salt samplers were reduced, and the bands for high salt samples remained nearly as intense as before filtration. These results are consistent with the concept that high salt prevents large particle sizes, low salt samples consist almost entirely of large particles, and intermediate salt produces a mixture of large and small particles. In fact, Figure 3a seems to indicate that intermediate NaCl concentration produces a small number of very large particles, that are larger than the largest particles formed at low salt. One possible explanation for this observation is a sort of slow growth model: in low salt, the protein may quickly coalesce principally into 1000 nm aggregates that do not aggregate further, while the aggregates that form at medium salt are surrounded by additional smaller particles that can continue to be adsorbed by the aggregated particles, ultimately producing larger particles than seen at low salt. This continuous process would be consistent with the very high polydispersity observed for the 250 mM NaCl sample.

### 3.4. Effect of Filtration on DLS Measurements, CD Spectroscopy, and Secondary Structure Analysis

**Figure 4:**
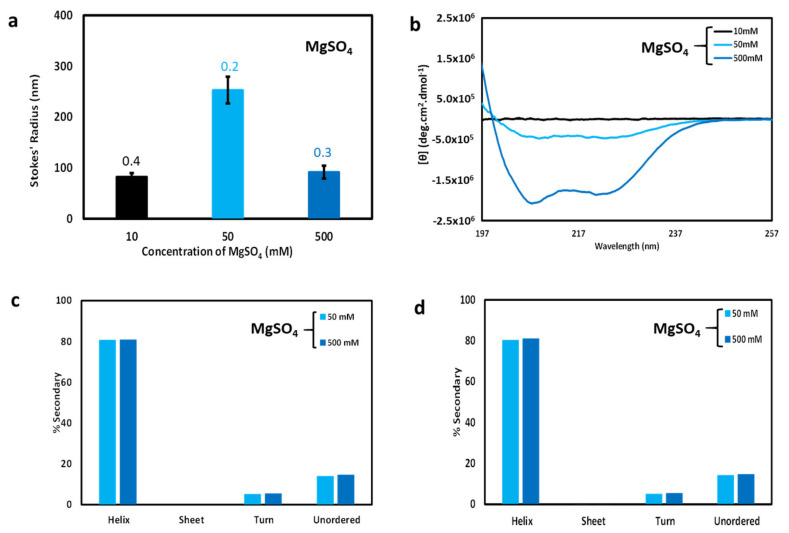
Effect of filtration on cl-Par-4 samples as a function of MgSO_4_ concentration. (**a**) DLS-derived Stokes’ radii after filtering. Error bars represent the standard deviation in Stokes’ radii from multiple runs, and the number above each bar represents the average polydispersity value for these runs. (**b**) CD spectra after filtration. (**c**) Secondary structure analysis via deconvolution of CD spectra from Figure 2b [unfiltered samples]. (**d**) Secondary structure analysis via deconvolution of CD spectra from Figure 4b [filtered samples]. Secondary structure analysis of unfiltered and filtered 10 mM MgSO_4_ samples was not possible due to the presence of large particles and low protein concentration, respectively.

To further explore the relationship between particle size and CD spectroscopy, filtered samples with low, medium, and high divalent cation concentrations (10, 50, and 500 mM MgSO_4_) were analyzed via DLS and CD spectroscopy. As expected, the hydrodynamic sizes derived from DLS measurements (Figure 4a) indicated smaller particle sizes relative to unfiltered samples (Figure 3b), except for the high salt 500 mM sample which predictably showed little change in particle size upon filtration. However, the filtered intermediate sample (50 mM MgSO_4_) produced the largest Stokes’ radius. This pattern for the filtered MgSO_4_ samples (Figure 4a) now resembles the pattern observed for the unfiltered NaCl samples (Figure 3a), with intermediate salt producing the largest particles, possibly due to a similar slow growth mechanism as was discussed in the previous section. Bear in mind, however, that very few particles of any sort survive filtration at low salt concentration.

CD spectra of the filtered samples are shown in Figure 4b. The filtered low MgSO_4_ sample predictably produced almost no dichroism, since the sample concentration after filtration was extremely low. As expected, the high MgSO_4_ sample produced a CD spectrum nearly matching the pre-filtered CD spectrum of Figure 2b. The most interesting result was that for medium MgSO_4_ (50 mM). Though the intensity was low relative to the filtered high salt sample, the shape nearly matches that of the high salt sample. This suggests that, although fewer particles were present in the filtered medium MgSO_4_ sample (see Figure 3d), and these particles were of relatively large size (see Figure 4a), the secondary structure was very similar to that of the particles present in high salt conditions. Thus, the difference in particle size found in the filtered medium and high salt samples does not correspond to a significant change in secondary structure. Apparently, cl-Par-4 particles can self-associate without a large change in secondary structure. However, this analysis only applies to particles small enough to survive filtration. Unfiltered samples with very large particles do produce differently shaped CD spectra (Figure 2b, low and medium salt samples).

Secondary structure analysis was performed via deconvolution of the CD spectra using the SELCON3 algorithm [26]. Deconvolution of unfiltered and filtered 50 mM and 500 mM MgSO_4_ spectra are shown in Figure 4c,d, respectively. The deconvolution program was unable to distinguish secondary structure differences between high and medium MgSO_4_ samples, before and after filtration: in each of these four cases, deconvolution suggests approximately 80% helix, 15% disorder, and 5% beta turn. Deconvolution of the unfiltered 10 mM salt CD spectrum suggested approximately 55% helix, and corresponding increases in the percentage of beta sheet, beta turn, and disorder. However, scattering by the large particles present in this sample would be expected to interfere with CD spectroscopy and hence with spectral deconvolution, and therefore the 10 mM MgSO_4_ secondary structure deconvolution is not presented in Figure 4c [33]. Deconvolution of the filtered low salt CD data was not possible due to lack of sufficient sample surviving filtration (see Figure 4b) and thus filtered low salt secondary structure is not presented in Figure 4d.

Both the filtered medium and high salt samples produce CD spectra with a θ_222_: θ_208_ ratio less than one. This was consistent with the presence of mostly non-coiled coil helices in both cases. Note that before filtration, the medium salt sample produced CD spectra with a θ_222_: θ_208_ ratio greater than one. This result suggests that the large aggregates present in the mixed-size environment of the unfiltered medium salt sample are responsible for the shifting of the θ_222_: θ_208_ ratio, and that particles small enough to survive filtration contain mostly non-coiled coil helices.

### 3.5. Effect of Centrifugation and Reintroduction of Salt

**Figure 5:**
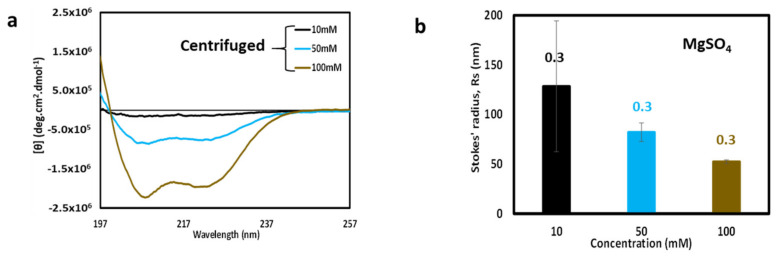
CD spectra and DLS analysis after centrifugation. (**a**) Overlay of CD spectra of supernatant after centrifugation of cl-Par-4 at various concentrations of MgSO_4_. The salt concentrations are indicated by the color legend within the panel. (**b**) Corresponding DLS of samples from panel (**a**). Error bars represent the standard deviation in Stokes’ radii from multiple runs, and the number above each bar represents the average polydispersity value for these runs.

To corroborate the results obtained from filtration, parallel experiments were performed by centrifuging the unfiltered samples and collecting the supernatant for CD analysis. CD spectra obtained from centrifuge supernatant samples are shown in Figure 5a. By comparison to the data of Figure 4b, it can be seen that filtration and centrifugation produced a similar effect of removing large aggregates, leaving smaller, highly helical particles in solution, although fewer particles survived filtration in the case of centrifugation. DLS analysis of the centrifuged samples used for the CD spectra in Figure 5a is shown in Figure 5b. The Stokes’ radii of each of the centrifuged samples confirm that only relatively small particles survive centrifugation.

**Figure 6:**
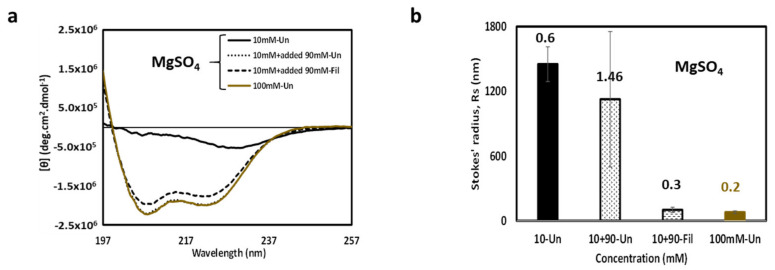
CD spectra and DLS analysis after reintroduction of salt. (**a**) 10 mM MgSO_4_ sample (solid black line) shows signs of large species formation (loss of signal); This signal loss can be eliminated by increasing the MgSO_4_ concentration to 100 mM (dotted black line); the resulting spectrum overlaps nearly perfectly with that of the original 100 mM sample (solid brown line). The dashed black line represents the slightly reduced CD spectrum of the added MgSO_4_ sample after filtration to remove any remaining large particles. (**b**) Corresponding DLS of samples from panel (**a**). Error bars represent the standard deviation in Stokes’ radii from multiple runs, and the number above each bar represents the average polydispersity value for these runs.

Also, to investigate the reversibility of large particle formation by cl-Par-4, 90 MgSO_4_ was added to low salt (10 mM MgSO_4_) samples. CD analysis showed that the resulting 100 mM MgSO_4_ spectrum becomes nearly identical to the original 100 mM MgSO_4_ sample which had never been at low salt (Figure 6a). Filtering of the resulting sample caused a small reduction in CD intensity, indicating the presence of a small number of large particles before filtration.

DLS analysis of the samples from Figure 6a is shown in Figure 6b. From left to right, the large detected Stokes’ radius at 10 mM MgSO_4_ appeared to become slightly smaller once 90 mM MgSO_4_ was added, but the large error bar and high polydispersity indicated a wide range of particle size. After filtration, the Stokes’ radius was much smaller, approaching that of the sample that had never been at low salt. The fact that filtration had a much larger effect on the DLS result than on the CD result indicates that the reconstituted 100 mM MgSO_4_ sample contained a small number of large particles that dominate the scattering. Removal of the large particles by filtration revealed that most particles were small under these conditions.

### 3.6. Time-Dependent Characteristics of cl-Par-4 Samples

**Figure 7:**
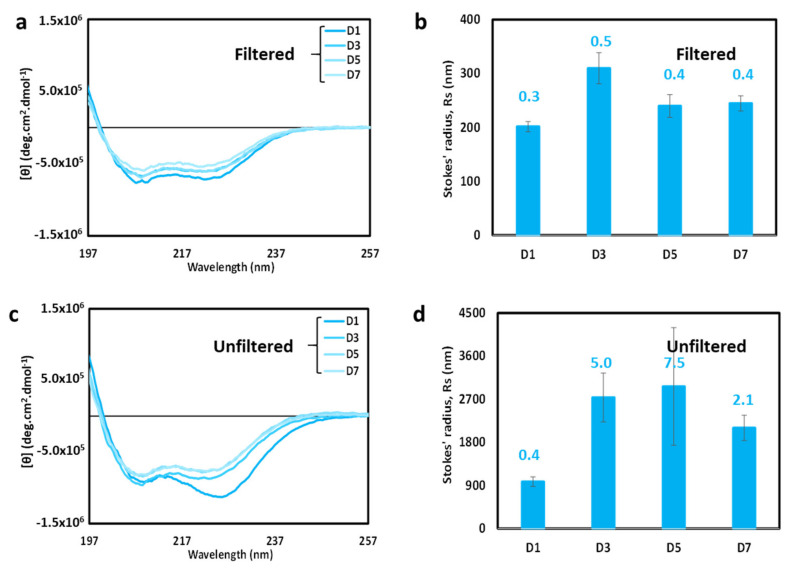
Time-dependence of cl-Par-4 in the presence of 50 mM MgSO_4_. (**a**) Overlay of CD spectra obtained over a seven-day time course. Sample was filtered on day 1 prior to the first spectrum. (**b**) DLS analysis of sample from panel (**a**). (**c**) Overlay of CD spectra obtained over a seven-day time course. Sample was NOT filtered. (**d**) DLS analysis of sample from panel (**c**). In panels (**b**) and (**d**), error bars represent the standard deviation in Stokes’ radii from multiple runs, and the number above each bar represents the average polydispersity value for these runs.

To further explore the possibility of dynamic equilibrium, time course CD spectroscopy experiments (Figure 7) were performed with samples at an intermediate salt concentration (50 mM MgSO_4_). The spectra in Figure 7a,b were performed with a sample that had been filtered on day 1. The shape of the CD spectra of the filtered sample changed relatively little over a seven-day period (see Figure 7a), suggesting that the sample does not rapidly aggregate over time. However, there is some loss of intensity, suggesting either sample loss or the formation of a small number of aggregates. DLS results showed that particle sizes remained relatively small over the time course (Figure 7b), thus indicating that loss of CD intensity was most likely due primarily to sample loss.

The change in the unfiltered 50 mM MgSO_4_ sample with time was more pronounced. The CD spectrum (Figure 7c) changed shape to resemble the filtered sample by day 3. This suggests that large particles may be lost from the unfiltered sample relatively quickly, either due to dispersion into smaller particles or via settling or adhering to the sample container. Since the intensity did not increase with time, the latter mechanism of removal by settling/adherence appeared dominant, but both mechanisms may be in play. DLS results (Figure 7d) showed a significant increase in measured Stokes’ radius and dispersity until day 5, and then a reduction in size and dispersity on day 7. The maximum particle size, to which DLS is sensitive, remained relatively large throughout the time course, indicating that aggregates were present throughout. However, since the CD spectra (Figure 7c) converted over time to a shape consistent with that displayed by smaller helical particles, the combined results seems to indicate that a small number of large particles remain over time, sufficient to influence the DLS results, but not sufficient to affect the CD spectra. The overarching result is that unfiltered, filtered, and centrifuged samples converge overtime to produce similarly shaped CD spectra indicative of highly helical particles. Taken together, these data are consistent with the presence of a dynamic equilibrium between small and large particles, which readjusts the following filtration, and is affected by settling/adherence.

## 4. Discussion

Full-length Par-4 (FL-Par-4) has been shown to be a largely intrinsically disordered protein (IDP) in vitro: CD spectroscopy and other techniques have previously been used to show that the C-terminal coiled-coil folds in FL-Par-4, while a large majority of the remainder of the protein maintains a disordered state under neutral conditions [34]. Caspase-3-induced cleavage of FL-Par-4 to form the N-terminal 15 kilodalton PAF fragment and the C-terminal 25 kilodalton cl-Par-4 fragment is necessary for the migration of cl-Par-4 to the nucleus, where it inhibits NF-κB-mediated cell survival pathways [13,14]. The PAF fragment has been shown to function as a decoy that protects FL-Par-4 from ubiquitin-mediated degradation [35].

We have previously shown that cl-Par-4 forms small, well-folded particles with approximately 80% helical content under either high sodium or low pH conditions [23,24]. There are differences, however. High sodium results in a tetramer conformation with a Stokes’ radius of approximately 100 nm, in which a significant portion of the helical content is not coiled coil. In contrast, acidic pH results in particles of a smaller size (approximately 50 nm Stokes’ radius), suggestive of a dimer, which does display coiled-coil character. Here we have shown that potassium and sodium have similar effects on cl-Par-4 structure (see Figure 2a). Thus, we can state that highly helical, significantly non-coiled particles, consistent with the Stokes’ radius of a tetramer, are also formed at high potassium concentration. This is a potentially meaningful finding, since potassium levels are higher inside of cells (of the order of 100 mM vs. 10 mM inside vs. outside cells), and sodium levels are higher extracellularly (of the order of 10mM and 100 mM inside vs. outside cells) [36,37]. This result suggests that cl-Par-4, which is found both intra- and extra-cellularly, may behave conformationally similarly inside and outside of the cell.

The amount of sodium or potassium necessary to induce the formation of these small helical particles appears to be higher than is typically encountered within either mammalian cellular or extra-cellular environments. In part for this reason, we investigated whether divalent cations could more efficiently induce this conformation. The results herein show that this is indeed the case, with 100 mM MgSO_4_ inducing the formation of relatively small, highly helical particles, at concentrations approximately five times lower than the required concentrations of sodium or potassium (see Figure 2c and Figure 3a,b). Together with previously published data identifying the conformation at high sodium concentration as a tetramer [24], this strongly suggests that tetramer is formed at the 100 mM MgSO_4_ concentration. The nearly identical results between MgSO_4_ and MgCl_2_ (see Figure 2d) also show that anion identity is far less important than cation identity for affecting cl-Par-4 conformation. The required magnesium concentration is still high relative to typical intra-cellular magnesium levels [36]. However, it has been reported that apoptosis is accompanied by alteration of cellular ionic conditions, including sodium, potassium, magnesium, chloride, calcium, and zinc, and changes in pH [36,38,39,40,41]. Thus, the combined effect of a number of factors should be considered, as they may conspire to affect the conformation of cl-Par-4 and other cellular proteins under less extreme conditions than are required of any one of these factors. Furthermore, the techniques employed in these studies require high micromolar protein concentration. Since in vivo cl-Par-4 levels will not reach high micromolar concentrations, cl-Par-4 should be less prone to aggregate and may not require as high ionic strength in order to form small, well-folded particles in vivo.

In support of this view, the present analysis of CD data, DLS data including polydispersity values, sample filtration, and gel electrophoresis, has shown that a mixture of particle sizes are present under various conditions. For intermediate conditions, large particles can be removed by filtration or centrifugation, and sufficient small particles remain for secondary structure analysis. These particles, which are sufficiently small to survive filtration, apparently have a secondary structure that resembles that of the small particles found at high salt (see Figure 4b). Thus, cl-Par-4 clearly can form highly helical structures under both low and medium salt conditions. This is at least true for particles that are not highly aggregated. Secondary structure analysis of the larger aggregates is complicated by the effects of scattering and will be discussed in more detail elsewhere.

As mentioned, approximately 80% helicity is observed in the small cl-Par-4 particles induced by sodium, potassium, or magnesium. The coiled-coil domain comprises only 38% of the cl-Par-4 sequence (see Figure 1a), thus additional helical regions are clearly present. Sequence analysis shows that the SAC domain, which comprises 28% of cl-Par-4, has the next highest order propensity (see Figure 1b). The linker domain has the highest disorder propensity. This strongly suggests that both the CC domain and at least a significant fraction of the SAC domain are helical in cl-Par-4 under these conditions. Previous biophysical analysis of FL-Par-4 provided no such evidence of folding of the SAC domain in the full-length protein [34] and suggested that the helical content in FL-Par-4 is largely coiled-coil in nature. Thus, cleavage of Par-4 by caspase-3 may be important both for altering the SAC domain conformation, and for influencing the self-association characteristics, including helical coiling, of the cl-Par-4 fragment in comparison to FL-Par-4. These two effects may in fact be correlated. It remains to be seen how conformational and self-association changes upon caspase-induced cleavage may be related to controlling the role of Par-4 in apoptotic processes.
